# Thyroid Hormone Signalling Genes Are Regulated by Photoperiod in the Hypothalamus of F344 Rats

**DOI:** 10.1371/journal.pone.0021351

**Published:** 2011-06-22

**Authors:** Alexander W. Ross, Gisela Helfer, Laura Russell, Veerle M. Darras, Peter J. Morgan

**Affiliations:** 1 Rowett Institute of Nutrition and Health, University of Aberdeen, Aberdeen, Scotland, United Kingdom; 2 Laboratory of Comparative Endocrinology, Division Animal Physiology and Neurobiology, Katholieke Universiteit Leuven, Leuven, Belgium; Vanderbilt University, United States of America

## Abstract

Seasonal animals adapt their physiology and behaviour in anticipation of climate change to optimise survival of their offspring. Intra-hypothalamic thyroid hormone signalling plays an important role in seasonal responses in mammals and birds. In the F344 rat, photoperiod stimulates profound changes in food intake, body weight and reproductive status. Previous investigations of the F344 rat have suggested a role for thyroid hormone metabolism, but have only considered *Dio2* expression, which was elevated in long day photoperiods. Microarray analysis was used to identify time-dependent changes in photoperiod responsive genes, which may underlie the photoperiod-dependent phenotypes of the juvenile F344 rat. The most significant changes are those related to thyroid hormone metabolism and transport. Using photoperiod manipulations and melatonin injections into long day photoperiod (LD) rats to mimic short day (SD), we show photoinduction and photosuppression gene expression profiles and melatonin responsiveness of genes by *in situ* hybridization; *TSHβ, CGA, Dio2* and *Oatp1c1* genes were all elevated in LD whilst in SD, *Dio3* and *MCT-8* mRNA were increased. *NPY* was elevated in SD whilst *GALP* increased in LD. The photoinduction and photosuppression profiles for *GALP* were compared to that of *GHRH* with *GALP* expression following *GHRH* temporally. We also reveal gene sets involved in photoperiodic responses, including retinoic acid and Wnt/ß-catenin signalling. This study extends our knowledge of hypothalamic regulation by photoperiod, by revealing large temporal changes in expression of thyroid hormone signalling genes following photoperiod switch. Surprisingly, large changes in hypothalamic thyroid hormone levels or *TRH* expression were not detected. Expression of *NPY* and *GALP*, two genes known to regulate GHRH, were also changed by photoperiod. Whether these genes could provide links between thyroid hormone signalling and the regulation of the growth axis remains to be investigated.

## Introduction

Many mammals and birds from temperate latitudes anticipate seasonal changes in climate and in response adapt their physiology and behaviour accordingly. These changes require resetting of a number of endocrine systems associated with reproduction, growth and energy balance, and through this strategy the species optimise their chances of survival for their offspring.

Recent work has demonstrated that the hypothalamus combines both role of photoperiodic time measurement as well as neuroendocrine regulator of physiology. [1]–[3]. Intra-hypothalamic thyroid hormone metabolism has been shown to play an important role in photoperiod-dependent seasonal responses. Pioneering studies by Yoshimura and colleagues have established the importance of hypothalamic thyroid hormone, T3, in the photoperiodic reproductive response of the Japanese quail [3]. More recent work in Siberian hamsters demonstrated that intrahypothalamic Silastic implants of T3 promoted long day-like reproductive and body weight responses in short day housed animals [2]. This suggested that thyroid hormone metabolism within the hypothalamus is important to photoperiodic regulation in mammals as well as birds. Furthermore in mammals, melatonin produced in the pineal gland was recently shown to act on the *pars tuberalis* (*PT*) to relay the photoperiod signal into the hypothalamus via the thyroid hormone signalling system. In long day housed sheep, melatonin-responsive cells in the PT increase production of thyrotrophin (TSH) relative to short day levels [1], a process recently shown to be coordinated by the photoperiod-responsive transcription factor Eya3 [4]. Thus TSH acts locally within the hypothalamus, through stimulation of the TSH-receptor expressing cells, a response also seen in the quail [5]. This leads to up-regulation of expression of type II deiodinase (Dio2), a key enzyme controlling thyroid hormone bioactivity. Dio2 converts thyroxine (T4) into the bioactive tri-iodothyronine (T3) in TSH-receptor (TSH-R) expressing cells located in the tanycytes of the ependymal layer of the hypothalamus. Increased expression of Dio2 should increase T3 availability within this region. In the quail, decreased expression of the T3 catabolizing enzyme, type 3 deiodinase (Dio3) has been reported in long days [6] which should reduce the clearance of active T3 in long days. Following a switch in photoperiod from long day (LD) to short day (SD), Dio2 decreases whereas the expression of Dio3 increases, which should produce a net decrease in hypothalamic T3 levels in SD. Consistent with this, the hypothalamic levels of T3 were shown to be ten fold lower in SD than LD whilst plasma levels were similar in both photoperiods in the quail [3]. Similar changes in *Dio2* gene expression occur in mammalian species such as the Soay sheep, Syrian hamster, the photoperiodically sensitive Fischer 344 (F344) rat, and mice [1], [2], [7]–[9]. The Siberian hamster differs in that only *Dio3* changes with photoperiod whilst no *Dio3* was observed in the ependymal cells in the Syrian hamster [2]. Despite this, it can be anticipated that the net effect of the changes in species including the Siberian hamster, would be high levels of hypothalamic T3 in LD and low levels during SD. Consistent with this, studies in the Siberian hamster have shown that central thyroid hormone metabolism plays a critical role in the seasonal control of body weight and reproduction [2].

The F344 rat also shows profound reductions in food intake and body weight in response to SD [10]. Previous investigations of hypothalamic genes involved in the food intake and body weight response to altered photoperiod in F344 rats demonstrated marked, but opposite changes in neuropeptide Y (*NPY*) and agouti-related peptide (*AgRP*) expression in the arcuate nucleus (ARC) [11]. It was postulated that upregulation of *AgRP* in LD was associated with the higher levels of food intake, whereas upregulation of *NPY* in the SD was associated with a reduced drive for growth. Study of the thyroid hormone signalling system in response to photoperiod in F344 rats has been limited to consideration of *Dio2* expression, which showed lower levels in SD than LD [9]. The relationship between intra-hypothalamic thyroid hormone signalling and body composition has not been investigated.

In this study we examined changes in hypothalamic gene expression in F344 rats in response to altered photoperiod by microarray. Amongst the most robust changes are genes related to pituitary/hypothalamic thyroid hormone responses. These changes were confirmed by *in situ* hybridization. In addition we investigated the dynamics of both photoperiod induction of gene expression using short to long day transition as well as photoperiod suppression, using transitions from long to short day and melatonin injections to mimic short day responses in long day housed rats.

We also identified changes in response to photoperiod of a number of other gene sets, including retinoic acid signalling genes but these are not the subject of this paper.

## Results

### TRH and thyroid hormone levels in F344 rats on LD and SD for 28 days

**Figure 1:**
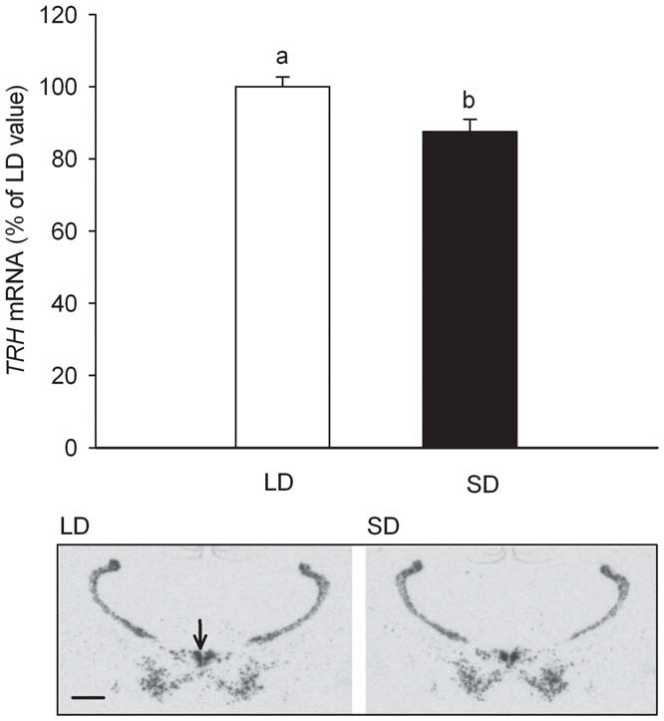
Effect of photoperiod on *TRH* gene expression in the paraventricular nucleus (PVN) of F344 rats. *TRH* mRNA levels in SD photoperiod are lower than in LD (*t*-test, *p* = 0.01). mRNA levels were determined by *in situ* hybridization (LD, n = 10; SD, n = 10). Data are mean ± SE. Autoradiographs of representative regions of coronal brain section images are shown for LD and SD. Arrow indicates PVN. Scale bar  = 1.0 mm.

Over a 28day period F344 rats held in SD (8 h∶16 h, light:dark) exhibited a slower rate of body weight gain (17.7% divergence, *p*<0.001) and reduced food intakes relative to rats held in LD (16 h∶8 h, light:dark) (previously shown in [11]). In brain sections from these rats, *TRH* mRNA expression in the paraventricular nucleus of the hypothalamus was modestly, but significantly suppressed in SD relative to LD rats after 28days (d) in the different photoperiods (SD 12.5% lower, *p*<0.01, Figure 1).

### Microarray of hypothalamus from LD and SD F344 rats

To explore how signalling through thyroid hormone-related pathways may have been affected at earlier times points, gene expression was compared using Affymetrix microarray of the hypothalamus taken from LD and SD rats after 3, 14 and 28d in the respective photoperiods, with follow up *in situ* hybridization on candidate genes.

**Figure 2:**
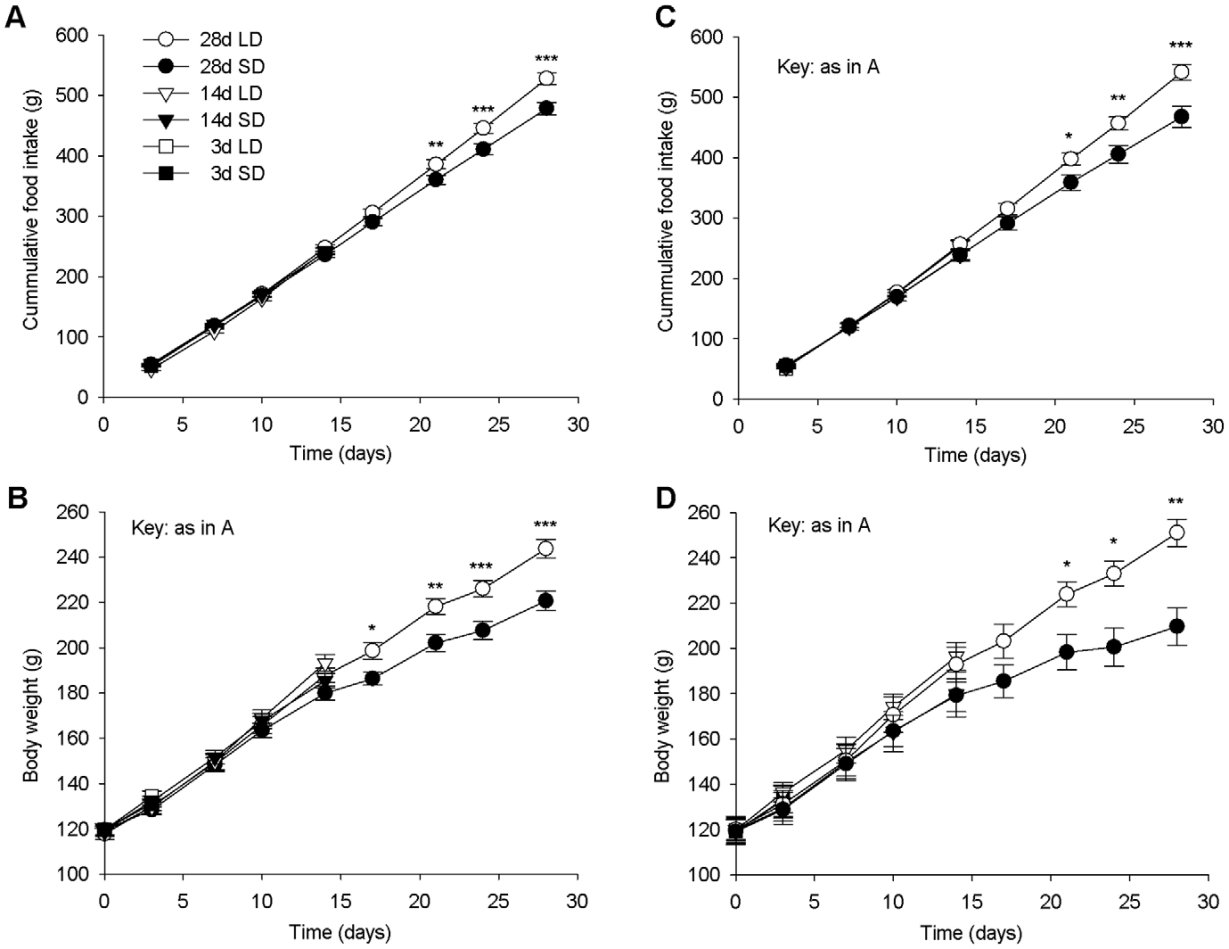
Photoperiod differentially affects food intakes and body weight gain of F344 rats. (A) Cumulative food intakes of SD rats used for *in situ* hybridization were significantly lower than LD rats after 21 days. (B) Body weights of rats used for *in situ* hybridization were significantly lower in SD compared to LD rats after 17d. (C) Cumulative food intakes of SD rats used for microarray were significantly lower than LD rats after 21 days. (D) Body weights of rats used for microarray were significantly lower in SD compared to LD rats after 21d. *, *p*<0.05; **, *p*<0.01 and ***, *p*<0.001.

Food intake and body weights at each of the time-points (3, 14 and 28d) of rats used for *in situ* hybridization and microarray are shown in Figure 2A–D. Food intakes were significantly lower in SD rats after 21d while body weights were lower after 17d in the rats used for *in situ* hybridization (n = 8 per group) but by 21d in the microarray groups (n = 4 per group). Total RNA from the hypothalamus, including the ARC, of F344 rats was analysed using the Affymetrix Rat 230-2 arrays. Genes that were either up- or down-regulated by 1.5 fold or more are shown in Tables 1–3. By microarray no significant change in TRH was seen, and *in situ* hybridization at the earlier time points 1, 3 and 14d also showed no significant difference in TRH mRNA expression with photoperiod.

**Table 1:** Genes found by microarray showing the largest responses to photoperiod after 3d.

Gene Name	Description	Fold Change	*p* value
**Genes down-regulated in SD**			
Hcrt	Hypocretin	−3.3	0.0441
Ccna2	cyclin A2	−1.8	0.0003
Gpr83	G protein-coupled receptor 83	−1.7	0.0027
RGD1563347	similar to RIKEN cDNA 2310015N21	−1.5	0.0117
Ces3	carboxylesterase 3	−1.5	0.0132
**Genes up-regulated in SD**			
Dao1	D-amino acid oxidase 1	1.5	0.0314
Slc6a4	solute carrier family 6 (5-HT transporter), member 4	1.5	0.003
LOC362972	similar to adiponutrin	1.5	0.0009
Tnnc2	troponin C type 2 (fast)	1.5	0.0241

**Table 2 pone-0021351-t002:** Genes found by microarray showing the largest responses to photoperiod after 14d.

Gene Name	Description	Fold Change	*p* value
**Genes down-regulated in SD**			
Tsh*β*	thyroid stimulating hormone, beta subunit	−243.96	2.52E-12
Gca	glycoprotein hormones, alpha subunit	−27.21	1.44E-05
Nmu	neuromedin U	−3.27	6.86E-08
Crabp1	cellular retinoic acid binding protein 1	−2.37	7.78E-05
Plunc	palate, lung, and nasal epithelium carcinoma associated	−1.79	0.0002
Ces3	carboxylesterase 3	−1.75	0.0019
Six1	sine oculis homeobox homolog 1 (Drosophila)	−1.65	0.0008
Sfrp2	secreted frizzled-related protein 2	−1.59	2.41E-05
Tnfsf13	tumor necrosis factor (ligand) superfamily, 13	−1.54	1.83E-05
Slco1c1	solute carrier organic anion transporter family, 1c1	−1.52	2.78E-05
Gup1	Gup1, glycerol uptake/transporter homolog (yeast)	−1.51	0.0199
**Genes up-regulated in SD**			
Ttr	transthyretin	9.32	0.0058
Foxg1	forkhead box G1	4.69	0.04
Bhlhb5	basic helix-loop-helix domain containing, class B5	2.34	0.0143
Hnrpdl	heterogeneous nuclear ribonucleoprotein D-like	2.14	0.0029
Ddn	Dendrin	2.08	0.0099
RGD1565710	similar to MGC68837 protein	2.08	0.0217
Neurod1	neurogenic differentiation 1	2.04	0.0338
Icam5	intercellular adhesion molecule 5, telencephalin	2	0.0038
Itpka	inositol 1,4,5-trisphosphate 3-kinase A	1.92	0.009
Slc17a7	solute carrier family 17 (Na-dependent inorganic phosphate cotransporter), member 7	1.9	0.0079
Lhx2	LIM homeobox protein 2 (predicted)	1.88	0.0263
LOC685826	similar to reprimo-like	1.84	0.0019
RGD1307524	similar to Friedreich ataxia region gene X123	1.83	0.0002
Gda	guanine deaminase	1.78	0.0196
Mlf1	myeloid leukemia factor 1	1.77	0.0009
Mx1	myxovirus (influenza virus) resistance 1	1.74	0.038
Kcnip2	Kv channel-interacting protein 2	1.7	0.0029
Lpl	lipoprotein lipase	1.65	0.002
RGD1566269	similar to Neuropilin- and tolloid-like protein 1	1.64	0.004
Myo5b	myosin 5B	1.57	0.0156

**Table 3:** Genes found by microarray showing the largest responses to photoperiod after 28d.

Gene Name	Description	Fold Change	*p* value
**Genes down-regulated in SD**			
Tsh*β*	thyroid stimulating hormone, beta subunit	−624.09	1.07E-13
Cga	glycoprotein hormones, alpha subunit	−32.88	6.84E-06
Nmu	neuromedin U	−5.72	7.16E-11
Crabp1	cellular retinoic acid binding protein 1	−3.16	1.72E-06
Rbp1	retinol binding protein 1, cellular	−2.84	3.53E-05
Stra6	stimulated by retinoic acid gene 6 homolog (mouse)	−2.53	2.68E-09
Ces3	carboxylesterase 3	−2.24	4.08E-05
Serpinf1	serine (or cysteine) peptidase inhibitor, clade F, member 1	−2.18	4.06E-05
Six1	sine oculis homeobox homolog 1 (Drosophila)	−2.17	4.86E-06
Aurkb	aurora kinase B	−2.16	1.10E-05
RGD1565710	similar to MGC68837 protein	−2.06	0.0237
Alas2	aminolevulinic acid synthase 2	−2.03	0.0122
LOC689064	beta-globin	−2.01	0.001
Olfml3	olfactomedin-like 3	−1.95	0.0015
S100g	S100 calcium binding protein G	−1.83	1.34E-05
Tnfsf13	tumor necrosis factor (ligand) superfamily, member 13	−1.74	4.98E-07
Ifitm1	interferon induced transmembrane protein 1	−1.73	0.001587
Timp1	tissue inhibitor of metallopeptidase 1	−1.73	0.000156
Sfrp2	secreted frizzled-related protein 2	−1.73	2.66E-06
Col1a2	procollagen, type I, alpha 2	−1.72	0.0046
**Genes up-regulated in SD**			
RGD1307524	similar to Friedreich ataxia region gene X123	1.79	0.0002
Tnnc2	troponin C type 2 (fast)	1.76	0.0016
NPY	neuropeptide Y	1.49	0.0236

Data from the microarray indicated that many genes showed differential expression between LD and SD at each time point but with most occurring at the 28d time point. A number of thyroid hormone signalling-related, retinoid signalling, Wnt/*β*-catenin signalling and energy metabolism-related genes were amongst those showing the greatest differences in expression in response to altered photoperiod. Several of these, together with some related genes that did not appear in the normalised array results, were analysed by *in situ* hybridization on coronal brain sections from rats that had been housed in LD or SD photoperiods over the same time course of 3, 14 and 28d, or 1, 3 and 14d.

**Figure 3:**
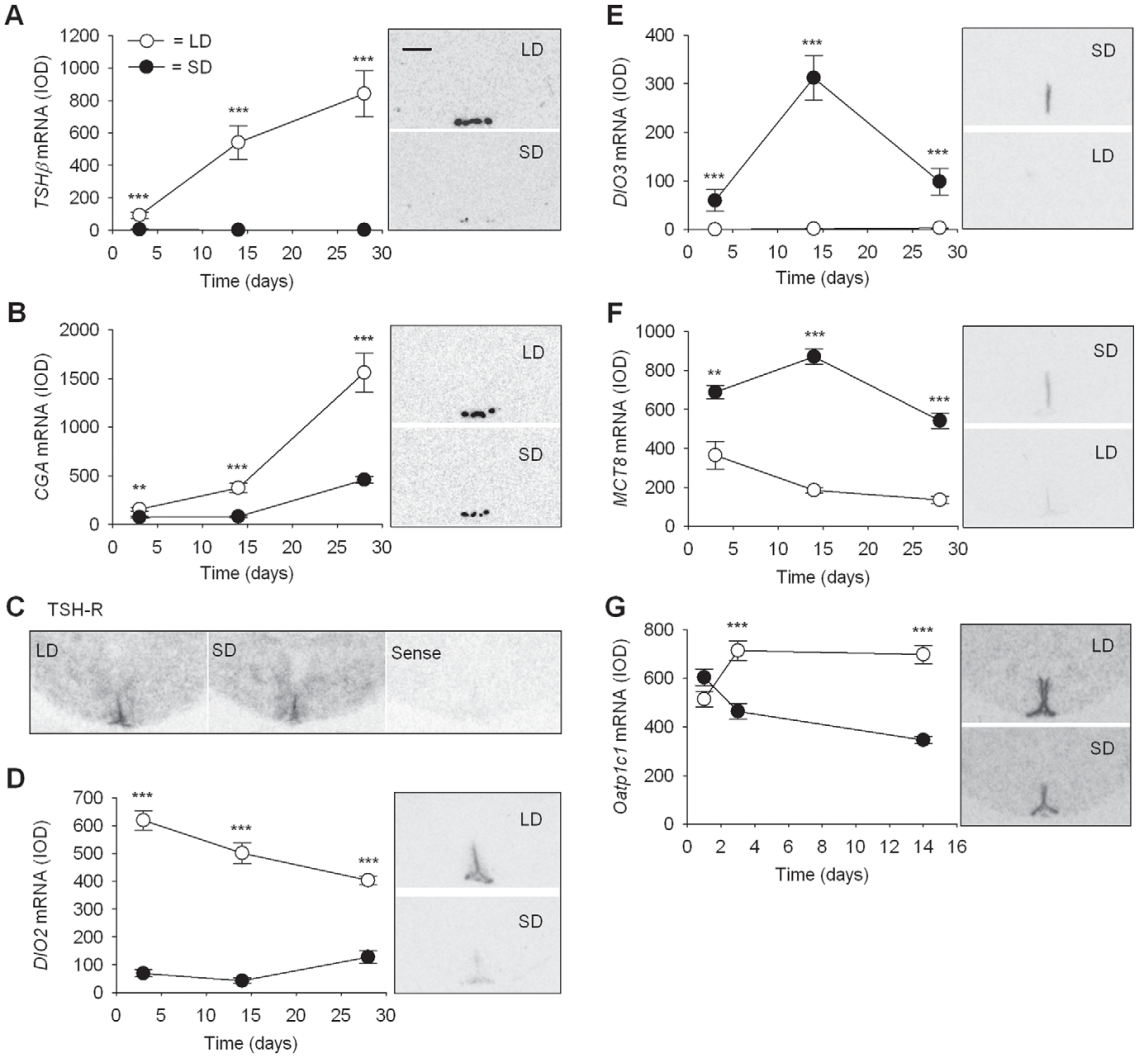
Effect of photoperiod on hypothalamic gene expression in F344 rats. (A, B, D and G) mRNA levels of *TSHβ* and *CGA (PT)* and *Dio2* and *Oatp1c1* (ependymal layer of hypothalamus 3^rd^ ventricle) are significantly greater in LD compared to SD photoperiods at time points from 3d onwards. (E, F) mRNA levels of *Dio3* and *MCT-8* (ependymal layer) are significantly higher in SD than LD photoperiods at each time point. (**, *p*<0.01; ***, *p*<0.001. Data are mean ± SE). Autoradiograph images of coronal brain sections adjacent to graphs show representative hybridization signals in the hypothalamus and *PT* for the same riboprobe at the time points of maximum difference in expression (scale bar  = 1.0 mm for all images). Images for *TSH-R* are representative antisense for LD (left) and SD (right) and include a typical sense image.

By microarray, gene transcripts for *TSHβ*and its binding partner *glycoprotein hormones, alpha polypeptide (CGA)* showed the largest differences in expression levels between LD and SD rats (Tables 2, 3). These results were confirmed by *in situ* hybridization; furthermore this analysis showed the expression of each gene to be restricted to the PT of the pituitary, with no expression observed in the hypothalamus (Figure 3A). Densitometric analysis showed that *TSHβ* levels were higher in LD than SD levels by 18.4 fold (*p*<0.001) after 3d, by 351 fold (*p*<0.001) after 14d and by 732 fold (*p*<0.001) after 28d (Figure 3A). Similarly *CGA* gene expression was expressed at higher levels in LD compared to SD rats at all time points, although unlike *TSHβ*, *CGA* expression levels increased over time in both photoperiods. The LD expression level was greater than the SD level by 2.1 fold (*p*<0.01), 4.8 fold (*p*<0.001) and 3.5 fold (*p*<0.001) at 3, 14 and 28d, respectively (Figure 3B).

Other genes related to TSH signalling that showed photoperiod-responsiveness by microarray included the receptor for TSH (*TSH-R*) and *Dio2*. These in addition to *Dio3* and a specific thyroid hormone transporter, *monocarboxylate transporter 8 (MCT-8)* were analysed by *in situ* hybridization over the time course. All of these genes were expressed in the mediobasal hypothalamic ependymal region around the 3rd ventricle in the F344 rat.

By microarray, there was a small but significant difference in expression of *TSH-R* between LD and SD at 3d, but at no other time points. Through *in situ* hybridization *TSH-R* was strongest in the ependymal region and the ventromedial hypothalamus but was not obvious in the PT, although the hybridization signal was too indistinct to allow densitometric analysis (Figure 3C).

In contrast, levels of *Dio2* mRNA, which was expressed in both the ependymal region and the mediobasal hypothalamus, were strikingly different at all time points with the highest expression observed in LD compared to SD after 3d. LD expression levels were greater than SD levels by 9 fold, 11.8 fold, and 3.2 fold at 3, 14 and 28d, respectively (all *p*<0.001, Figure 3D).

*Dio3* mRNA levels were also robustly influenced by photoperiod with a significant increase seen as early as 3d in the ependymal layer in SD. The levels in SD increased substantially to peak at 14d before decreasing again by 28d, but remaining higher than LD levels throughout. *Dio3* mRNA levels were up-regulated in SD by 270-fold at 3d, 210-fold at 14d and 30-fold at 28d relative to LD levels (all *p*<0.001, Figure 3E).

*MCT-8* mRNA expression in the ependymal region was higher in SD compared to LD at all time points. SD *MCT-8* levels increased relative to LD by 1.9-fold at 3d (*p*<0.01), 4.7-fold at 14d and 4.0 fold at 28d (both *p*<0.001, Figure 3F). In SD rats *MCT-8* expression peaked at 14d whereas in LD rats the levels steadily decreased with time (Figure 3F).

A further thyroid hormone transporter, organic anion transporter family member 1C1 *(Oatp1c1)*, identified as differentially expressed on the microarray (Tables 2,3), was also shown to be expressed in the ependymal region and median eminence by *in situ* hybridisation (Figure 3G). Densitometry showed that *Oatp1c1* was expressed at significantly higher levels in LD compared to SD at 3d and 14d (both *p*<0.001, Figure 3G) following photoperiod change, but not after 1d. This expression pattern contrasts markedly with *MCT-8*.

**Figure 4:**
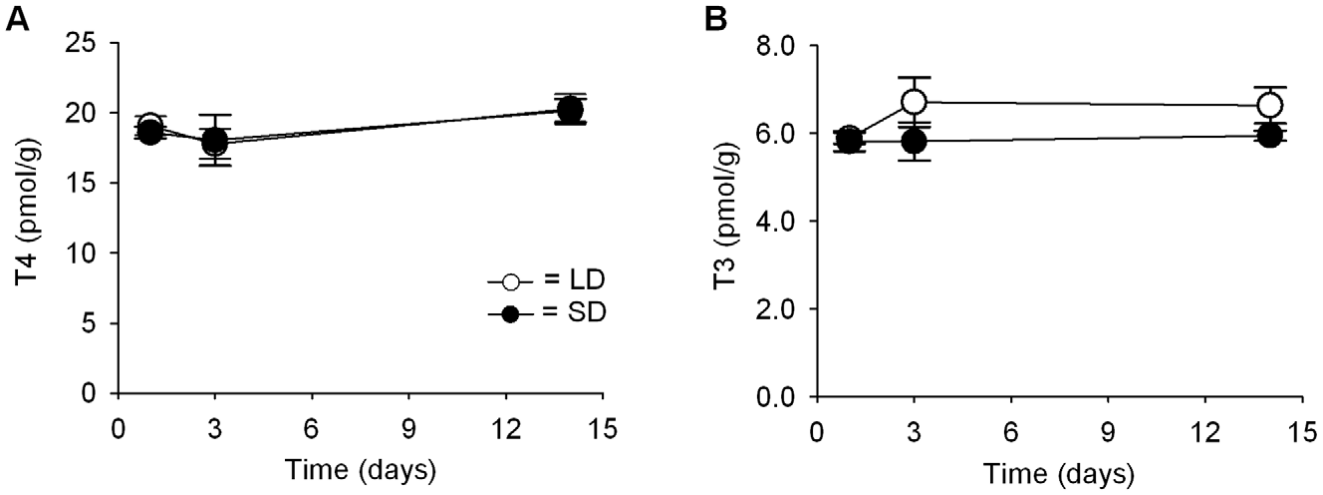
Thyroid hormone levels in hypothalamic tissue. (A) T4 levels show no change with photoperiod at any time point. (B) T3 levels are slightly but not significantly higher in LD than SD at 3 and 14d but not after 1d. Results are from radioimmunoassay and data are mean ± SE.

In view of the rapid changes in expression of genes associated with thyroid hormone signalling with all components significantly different within 3d following photoperiod change, the levels of thyroid hormones (T3 and T4) in the hypothalamus were assessed at the time points of 1, 3 and 14d following photoperiod switch. No significant difference in response to photoperiod change in T3 or T4 levels in hypothalamic tissue extracts at any of these time points was found, although the T3 levels tended to rise after 3d in LD compared to SD (Figure 4A,B).

### Effect of melatonin, photoinduction and photosuppression on thyroid hormone signalling genes

For the microarray experiments, the effects of either LD or SD were measured after switch from an intermediate photoperiod of 12 h∶12 h, light:dark, providing simultaneous photoinduction and suppression. To examine these two effects independently and to establish the role of melatonin, thyroid signalling responses (*TSHβ, Dio2, Dio3* and *Oatp1c1* gene expression) were examined after switch from either LD14∶10 (14 h∶10 h, light:dark) or SD10∶14 (10 h∶14 h, light:dark) photoperiods. These photoperiods were chosen because they have been used previously to demonstrate effectiveness of melatonin injections into LD10∶14 to mimic SD physiological responses [12].

**Figure 5:**
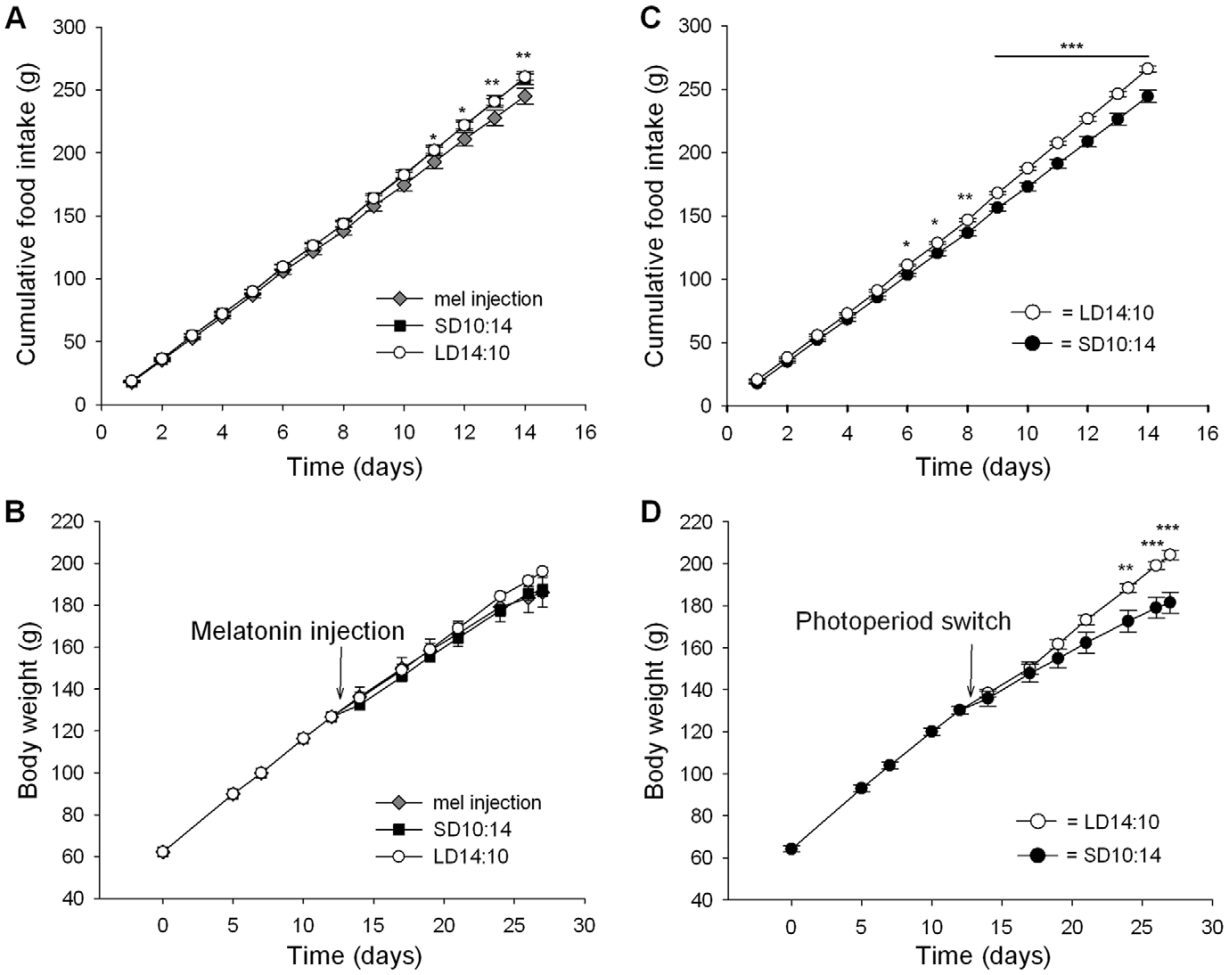
Body weights and food intakes of photosuppression, melatonin injection, and photoinduction rats. (A) Cumulative food intakes of rats injected with melatonin for 14d compared to LD14∶10 and SD10∶14 rats. Food intakes of melatonin injected rats are significantly lower than LD14∶10 control rats after 11d (*p*<0.001), but there is no difference between LD14∶10 and SD10∶14. (B) Body weights of rats injected with melatonin showed no significant difference after 14d of treatment. (C) Food intakes of rats following photoperiod switch from SD10∶14 to LD14∶10. The food intakes become significantly different 6d after the photoperiod change. (D) Body weights of rats in SD10∶14 and following photoperiod switch to LD14∶10 where the weights diverge significantly 11d after the switch. The point of photoperiod change is shown by the arrow (*, *p*<0.05; **, *p*<0.01; ***, *p*<0.001). Data are mean ± SE.

**Figure 6:**
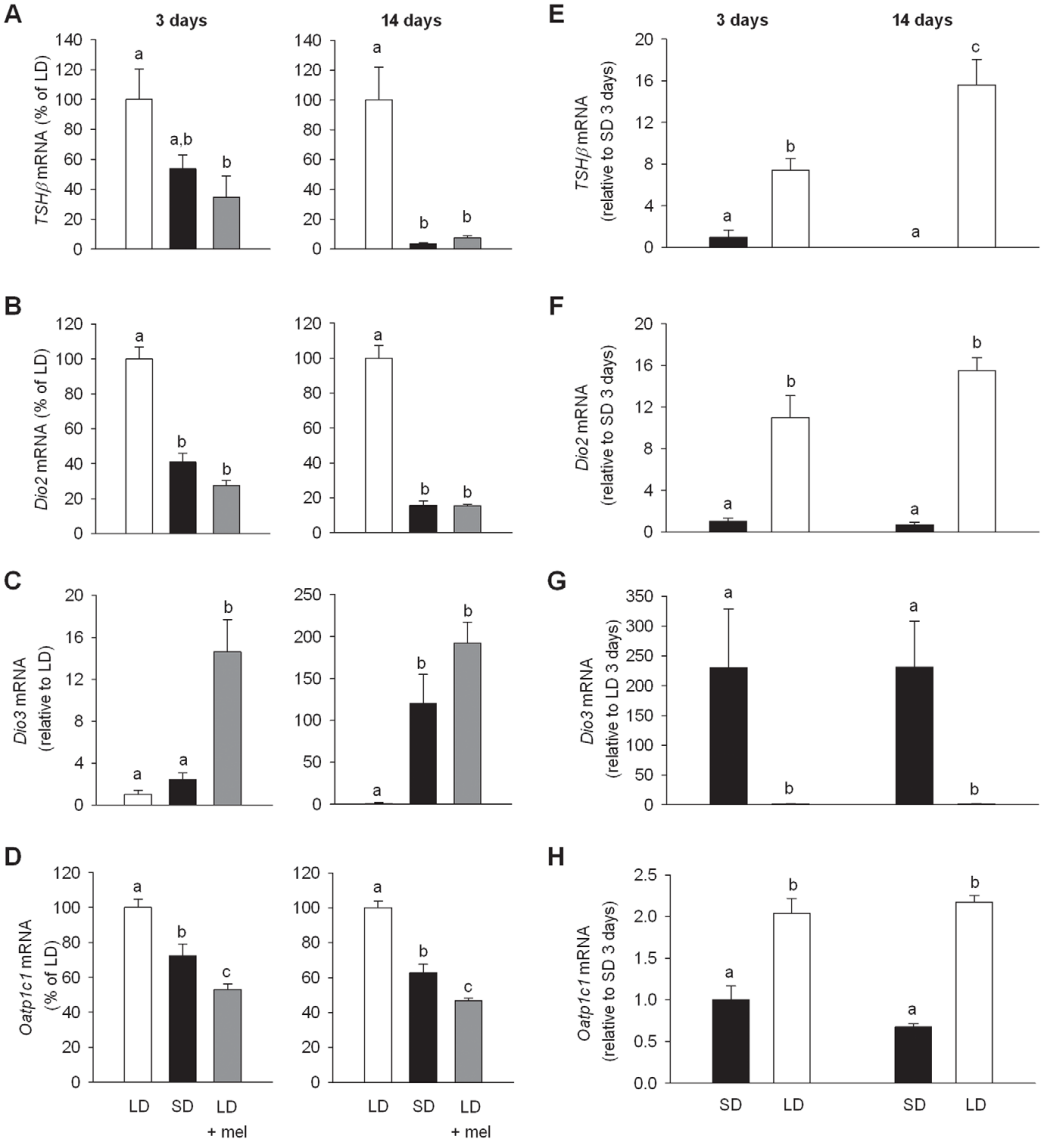
Effects of melatonin, photosuppression and photoinduction on thyroid hormone signalling genes. (A–D) Graphs are results of *in situ* hybridizations that show effects of SD10∶14 (SD) and melatonin injection into LD14∶10 (LD + mel) on *TSHβ, Dio2, Dio3* and *Oatp1c1* levels of gene expression respectively, compared to LD14∶10 (LD) at 3d and 14d time points. *TSHβ, Dio2* and *Oatp1c1* mRNA levels are suppressed by melatonin injection after 3d (*TSHβ p = 0.015*, *Dio2* and *Oatp1c1, p*<0.001) and suppressed further by 14d (*TSHβ*, *Dio2* and *OATP1c1,* all *p*<0.001). *Dio3* in contrast was induced by melatonin injection after 3d (*p*<0.001) and by both melatonin and SD10∶14 after 14d (*p*<0.001). (E–H) Graphs show the effects of transfer of rats from SD10∶14 to LD14∶10 on gene expression levels of *TSHβ, Dio2, Dio3* and *Oatp1c1* respectively, after 3 and 14d. *TSHβ, Dio2* and *Oatp1c1* gene expression are significantly induced whilst *Dio3* is inhibited at 3d (*TSHβ, Dio2* and *Oatp1c, p*<0.001; *Dio3 p*<0.015). For *Dio2*, *Oatp1c1* and *Dio3,* these responses were sustained at 14d (*Dio2* and *OATP1c, p*<0.001; *Dio3 p*<0.01) whilst the magnitude of the response for *TSHβ*was further increased by 14d (*TSHβ p*<0.001). Bars with different letters are significantly different. Data are mean ± SE.

To assess the effects of photosuppression and melatonin, F344 rats were divided into three groups. A control group was maintained on LD14∶10 whilst a second group was transferred to SD10∶14 and a third group was maintained on LD14∶10 and given a sub-cutaneous injection of melatonin one hour before lights off to extend the melatonin signal and thereby mimicking a short day response [12]. Food intakes were significantly lower in the LD14∶10-melatonin injected and SD10∶14 rats compared to the LD14∶10 control rats after 11d (*p*<0.001, Figure 5A). The body weights began to diverge but differences did not reach significance (Figure 5B) and paired testes weights also showed no significant differences (Figure S1). Gene expression for *TSHβ, Dio2, Dio3* and *Oatp1c1* after 3d and 14d of treatment are shown in Figure 6A–D. Relative to the LD14∶10 control group, gene expression of *TSHβ, Dio2* and *OATP1c1* was significantly suppressed after 3d of melatonin injections (*TSHβ p = 0.015*, *Dio2* and *Oatp1c1, p*<0.001), with the magnitude of the response being greatest after 14d (*TSHβ*, *Dio2* and *OATP1c1,* all *p*<0.001). Melatonin injections gave a comparable response to SD10∶14, both in magnitude and temporally. For *Dio3*, SD10∶14 induced a time dependent increase in gene expression only after 14d (*p*<0.001, Figure 6C), although melatonin induced a response of much greater magnitude at 3d, relative to the LD14∶10 level of expression (Figure 6C). *Dio3* induction was more pronounced under the 8 h∶16 h light:dark, SD photoperiod than in the SD14∶10 (compare Figure 3E with 6C).

In the photoinduction experiment where rats were transferred from SD10∶14 to LD14∶10, food intakes and body weights were significantly greater in LD14∶10 rats from 6d and 11d respectively following the switch, compared to SD10∶14 rats (*p*<0.001 for both. Figure 5C,D). Significant induction of *TSHβ, Dio2* and *Oatp1c1* and inhibition of *Dio3* gene expression were observed at 3d (*TSHβ, Dio2* and *Oatp1c1, p*<0.001, *Dio3, p*<0.015, Figure 6E–H). For *Dio2*, *Oatp1c1* and *Dio3*, these responses were sustained at 14d (*Dio2* and *Oatp1c1, p*<0.001; *Dio3, p*<0.01, Figure 6F–H) whilst the magnitude of the response for *TSHβ*was further increased by 14d (*TSHβ p*<0.001, Figure 6E).

### GHRH and potential regulatory genes (*GALP* and *NPY*)

**Figure 7:**
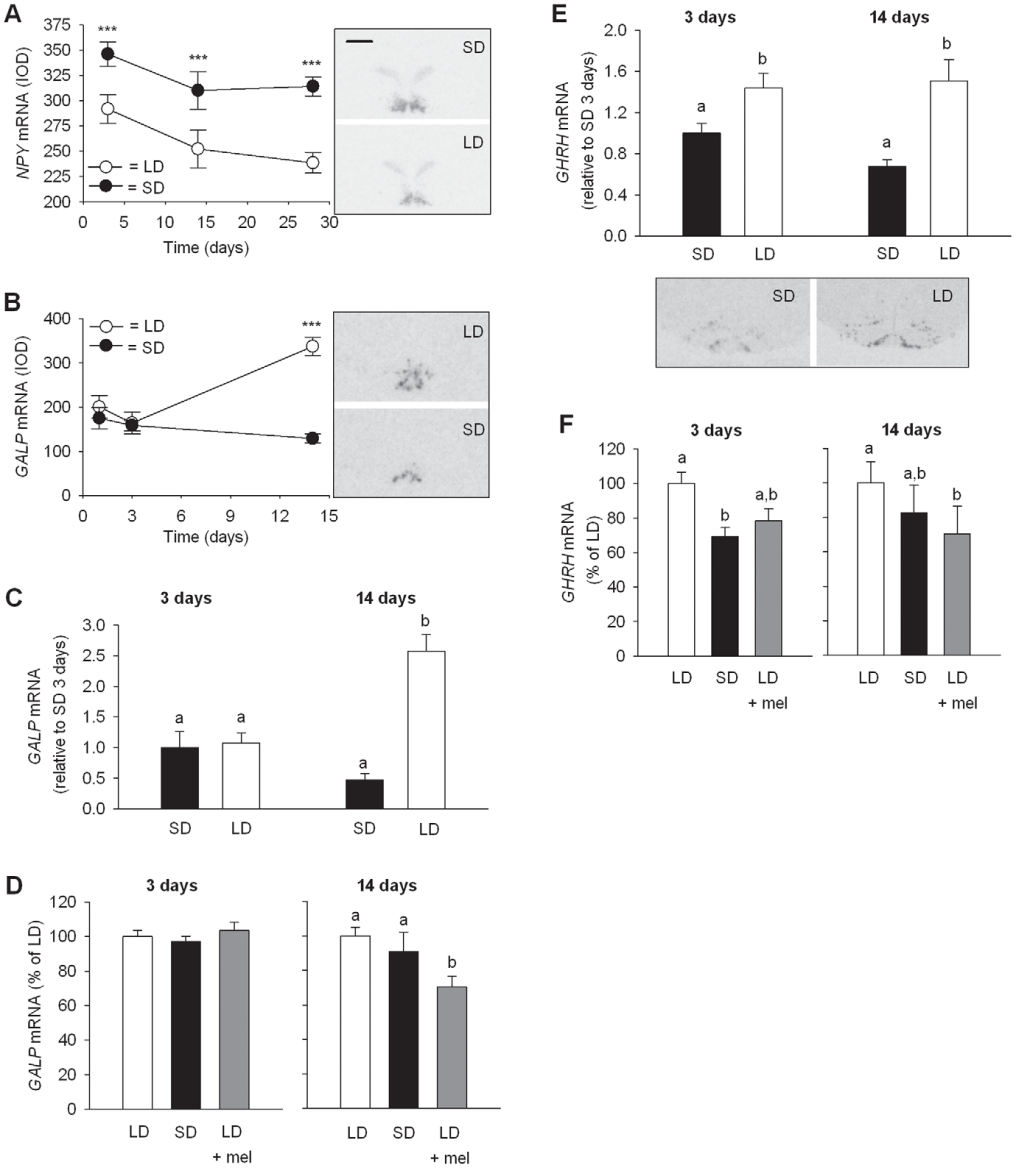
Comparison of effects of photoperiod on *NPY, GALP* and *GHRH*. (A) ARC *NPY* mRNA levels show decreased expression in LD compared to SD rats as early as 3d post photoperiodic switch, and the response is sustained over 28d (*p*<0.001 at all time points). (B) A significant increase in ARC *GALP* expression in LD compared to SD occurs only after 14d (*p*<0.001) but not at the earlier time points of 1 and 3 days. (C) The photoinduction paradigm shows *GALP* mRNA increases only after 14d of the photoperiod switch from SD10∶14 to LD14∶10 (*p*<0.001) with no change apparent after 3d. (D) *GALP* mRNA decreases significantly only after 14d of melatonin injections into LD14∶10 rats compared to control LD14∶10 rats (*p* = 0.038), but not after 3d or 14d in SD10∶14. (E) ARC *GHRH* expression changes significantly 3d (*p*<0.01) after photoperiod transfer and this was sustained after 14d (*p*<0.05). (F) In the suppression paradigm *GHRH* expression decreases significantly after 3d in SD10∶14 (*p*<0.01) but a decrease after 14d does not reach significance. Melatonin injections causes *GHRH* to decrease significantly only after 14d (*p*<0.01). Autoradiograph images of coronal brain sections adjacent to graphs show representative hybridization signals in the hypothalamus for the same riboprobe at the time points of maximum difference in expression (scale bar  = 1.0 mm for all images). Data are mean ± SE.

Two hypothalamic neuropeptides, differentially expressed on the microarray from LD and SD F344 rats, were *NPY* and *GALP*. Both genes have been implicated in the regulation of growth hormone releasing hormone (GHRH), and hence growth, and galanin-like peptide (GALP) is a potential target protein for thyroid hormone [13]–[15]. *GALP* expression by microarray increased significantly in LD compared to SD after 14d and 28d (−1.32, *p* = 0.004 at 14d; −1.39, *p* = 0.001 at 28d). In contrast, *NPY* mRNA increased significantly in SD compared to LD after 28d (1.49, *p*<0.02). By *in situ* hybridisation it was confirmed that both *NPY* and *GALP* genes are differentially expressed in the hypothalamus, and more specifically in the ARC (Figure 7A,B). However the temporal expression profiles were markedly different for the two peptides. *NPY* showed a rapid decrease in expression after only 3d in LD relative to SD; this differential expression was sustained over 28d (*p*<0.001 at all time points, Figure 7A). In contrast, the response of *GALP* to photoperiod change was much slower. A significant increase in *GALP* expression was seen in LD compared to SD only after 14d (*p*<0.001) but not at the earlier time points of 1 and 3 days (Figure 7B). A significant increase in *GALP* expression was also seen in the photoinduction paradigm only after 14d of the photoperiod switch from SD10∶14 to LD14∶10 (*p*<0.001) with no change apparent after 3d (Figure 7C). This time-course of change in *GALP* mRNA levels was also investigated in response to melatonin injections and in the photosuppression paradigm. *GALP* mRNA decreased significantly only after 14d of melatonin injections into LD14∶10 rats compared to control LD14∶10 rats (*p* = 0.038), but not after 3d or 14d in SD10∶14 (Figure 7D).

In response to photoinduction, *GHRH* expression in the ARC changed significantly after 3d (*p*<0.01) of treatment and was sustained after 14d (*p*<0.05, Figure 7E). In the suppression paradigm, *GHRH* expression decreased significantly after 3d in SD10∶14 (*p*<0.01) but the decrease after 14d was not statistically significant (Figure 7F). With melatonin injections, *GHRH* decreased significantly only after 14d (*p*<0.01, Figure 7F).

Other genes showing large changes in expression in LD compared to SD by microarray included the retinoic acid-related genes, *CRABP1*, *CRBP1* (Tables 2, 3) and the retinoic acid receptor *Stra6* (Table 3). Genes with functions related to appetite and energy balance regulation included *NPY* which was higher in SD than in LD, and *NMU* which was the opposite (Tables 2, 3). We recently described the expression of *CRBP1* and *Stra6* in the rat hypothalamus, and the SD induction of *NPY* [11], [16], and consideration of these and many of the other genes are outside the scope of this paper and will be the subjects of future studies.

## Discussion

This is the first study to examine the effects of photoperiod on gene expression in the mammalian hypothalamus, using the F344 rat as a model. A wide range of differentially expressed genes was identified, but with three prominent gene sets being revealed: 1. those involved in thyroid hormone signalling, transport and metabolism; 2. those involved in vitamin A/retinoic acid signalling and 3. those involved in Wnt/*β*-catenin signalling. In this study we have focussed on the thyroid hormone signalling-related gene set with the other gene sets being the subjects of other studies.

Of the thyroid-related signalling genes, *TSHβ* and *CGA*, expressed in the *PT*, exhibited the biggest difference in expression between long and short photoperiods. These findings accord with those from other species [1], [5]. Non-covalent bonding of the *TSHβ* and *CGA* subunits is required for biological activity of TSH [17] and hence the presence and changes in the two subunits suggests that there is functional TSH production. The presence of the TSH receptor is important for the central TSH signalling pathway to function as reported for other species, and here we detected expression of the *TSH-receptor* in the F344 rat ependymal region. In sheep it has been shown that the downstream response to TSH-receptor activation in this region involves an increase in cAMP production, and in quail, sheep and Syrian hamsters includes the LD activation of Dio2 to generate active T3 [1], [2], [5], [8], [18]. In our experiments, *Dio2* mRNA was induced time-dependently by LD in the F344 rat whereas it was suppressed in SD10∶14 and LD14∶10 melatonin injected rats. The time-course of *Dio2* gene induction or suppression under LD and SD seems to correlate well with the time-frame of *TSHβ* expression, suggesting a functional linkage. Taken together the data from this study provide further evidence in support of the consensus from quail, sheep and mice for retrograde signalling by PT derived TSH in the hypothalamus providing photoperiodic control of signal transduction and gene expression in the ependymal region, specifically *Dio2* [1], [5], [7].

There is one difference of note, which is the contrast between the temporal dynamics of expression of *TSHβ* in the rat in this study and those results for the Syrian hamster. In the latter, suppression of *Dio2* by SD was shown to occur ahead of a change in *TSHβ* [19]. On this basis it has been hypothesised that for Syrian hamsters, TSH*β*is involved in the induction phase of the photoperiod response only, and that suppression of this system may require a different, unknown mechanism [19], [20]. In this study, as early as 3d following either melatonin injections into LD14∶10 rats or SD10∶14 treatments, levels of *TSHβ* and *Dio2* gene expression were similarly inhibited. Likewise during photoinduction from SD10∶14 to LD14∶10, the time-course of increased gene expression for *TSHβ* and *Dio2* was comparable. Thus there is no evidence of temporal dissociation of *TSHβ* and *Dio2* gene expression for either photoinduction or suppression in the rat. In this study, our data suggest that the SD photosuppression is most likely due to photoperiod-driven melatonin production.

As in other species, *Dio2* is not the only thyroid hormone metabolising enzyme under photoperiodic control in the F344 rat. *Dio3*, a gene encoding an enzyme regulating T3 inactivation, was strongly induced in SD within 3d, peaking at 14d. This response was stimulated by melatonin injections into LD14∶10 rats with a similar time-course. Once again similar *Dio3* responses to SD have been observed in other species [1], [2], [6], [7]. Acting in concert with *Dio2*, these changes in *Dio3* would be predicted to cause an increase in local ependymal T3 concentrations in LD and a fall in SD as seen in the quail [3]. Surprisingly, in this study only a small rise in T3 after 3d was observed, which failed to reach statistical significance.

One potential explanation for this disparity between the profound changes in gene expression of the deiodinases and the relatively unchanged hypothalamic thyroid hormone levels may be local, dynamic changes in thyroid hormone transporters that can rapidly regulate thyroid hormone levels. Monocarboxylate transporter, *MCT-8* was found to be strongly expressed with higher levels in SD than LD in the ependymal region of the hypothalamus. Cells containing this transporter are thought to rapidly equilibrate thyroid hormone levels with the ability to import as well as release thyroid hormones [21]. Elevated *MCT-8* mRNA levels were also observed in Siberian hamsters under SD and it has been proposed that these may be involved in the decline in active hypothalamic T3 [22]. A second thyroid hormone transporter, *Oatp1c1*, was found by microarray and confirmed by *in situ* hybridization to be strongly expressed in the ependymal region, with sustained higher levels in LD compared to SD. *Oatp1c1* mRNA was also found to be responsive to melatonin injections in LD14∶10 rats, which mimicked the SD response. This transporter has been reported to transport T4 from the CSF into endothelial cells with higher affinity than T3 and also mediates bidirectional transport [23]. In cells where Oatp1c1 and deiodinases are co-expressed, Oatp1c1 increases access to the deiodinases, greatly increasing substrate metabolism, thus indicating that Oatp1c1 expression can be rate limiting for iodothyronine metabolism by the deiodinases [24]. However, the properties and role of Oatp1c1 in the photoperiodic mammal appear to be slightly different from those in chicken due to differences in substrate preference [25]. Also cOatp1c1 was found to be present in abundance in the ependymal cells lining the ventro-lateral walls of the third ventricle, yet there was no detectable change in chicken mRNA expression with photoperiod [25]. Thus, the local rapid equilibration of thyroid hormone levels possible with these transporters, which differ with photoperiod, may account for the difference in the level of detectable thyroid hormone in the rat compared to the quail [3]. These results highlight not only that there may be differences in the regulation of thyroid hormone transport and metabolism between species, but also show how important it is to gain a complete picture of all the players and components regulating thyroid bioavailability in the hypothalamus so as to fully understand their relevance to physiological function. It also remains a possibility that there could be a phase angle difference in daily rhythmicity in the thyroid hormone and *TRH* responses.

While the lack of large changes in hypothalamic T3 and T4 in this study, in contrast to quail [3], may seem surprising in view of the profound changes in gene expression of thyroid hormone related genes, there are physiological reasons why sustained perturbed changes in T3/T4 may not be expected. Thyroid hormones in the hypothalamus are known to feedback and inhibit TRH to provide a tightly regulated set-point of TRH and the thyroid endocrine axis [26], [27]. The unchanging hypothalamic thyroid hormone levels observed in this study and the modest change in *TRH* mRNA in the paraventricular nucleus are consistent with this model of set-point control. Nonetheless, it should be noted that photoperiod was not without some effect on *TRH* gene expression, causing a slight decrease in SD, indicating some effect of photoperiod on TRH neurons.

Overall, the players necessary for the transduction of photoperiodic information, from the induction of *TSHβ* and *CGA* in the *PT*, through retrograde signalling in to the hypothalamus to adjust deiodinase activities and thereby modulate local thyroid hormone signalling, all appear to be in place in the F344 photoperiodic rat, as described in other species. The lack of detectable thyroid hormone change may simply be because it is a transient event that is rapidly equilibrated after the downstream output is activated, emphasising the need to investigate hormone flux rather than static levels. These thyroid hormone-dependent changes are thought to impact on seasonal reproduction and body weight [1]–[3]. However, the output from this signalling system remains to be identified.

One of the genes identified on the microarray as differentially expressed between LD and SD rats, and a potential target of triiodothyronine and intermediate between thyroid hormone signalling and GHRH is *GALP*. GALP was originally discovered as a ligand of galanin receptors in the porcine hypothalamus [28]. GALP distribution in the rat brain is predominantly in neural cell bodies in the hypothalamic ARC, median eminence and infundibular stalk [29], [30]. *GALP* expression may be regulated by thyroid hormone, given that thyroidectomized rats have been reported as having significantly fewer *GALP*-expressing cells in the ARC than sham operated controls, and replacement of thyroxine partially reverses this effect [14]. GALP is also regulated by leptin and insulin and is thought to participate in the regulation of reproduction and metabolism [31] and may play a role in growth regulation. GALP can stimulate growth hormone secretion in Rhesus monkey and rat [32], [33]. In addition, recently, a role for GALP in stimulating intracellular calcium concentrations in GHRH neurons in the ARC, but not in NPY or POMC neurons, has been reported [13]. GHRH is the main stimulatory neuropeptide involved in generating and maintaining GH secretion in mammals [34].

Previously we showed that *GHRH* mRNA levels in the ARC were higher after 28d in LD than SD in the F344 rat [11] and we show here that the SD suppression could be achieved within 3 days. The reciprocal up-regulation of *GHRH* was observed within 3 days of transfer from SD10∶14 to LD14∶10. *GALP* however, was found to be expressed in the arcuate nucleus of the F344 rat but did not show higher levels of mRNA expression in LD compared to SD until 14d. Although the *GALP* mRNA expression seen in LD14∶10 rats was also attenuated in melatonin injected LD14∶10 rats after 14d, there was no significant difference between LD14∶10 and SD10∶14 rats at 3 or 14d.

At present there is insufficient evidence to invoke GALP as an intermediate between photoperiodically controlled thyroid hormone signalling and GHRH, due to the temporal asynchrony between the changes in *GALP* and *GHRH* gene expression, such that *GHRH* mRNA responses occurred ahead of any changes in *GALP* mRNA level with either photoperiod or melatonin. This may suggest that GALP is not the primary photoperiod-driven regulator of GHRH, or that it may be involved in sustaining the longer term GHRH changes. Also at present we cannot exclude whether other mechanisms, such as translational or secretory control of GALP may provide routes to the regulation of GHRH release. Previously we have suggested that NPY may provide a mechanism through which *GHRH* expression may be regulated [11]. The rapid increase in *NPY* gene expression in SD versus LD rats, 3d post photoperiod switch, without any associated change in food intake, suggests that, in this context, NPY serves a different function to the regulation of appetite. As the time-course of gene expression changes in *NPY* are comparable to *GHRH*, the potential for NPY to act as a regulator of GHRH remains a possibility [15].

## Methods

### Ethics Statement

All animal experiments were performed under strict adherence to UK home office regulations according to the Animals (Scientific Procedures) Act, 1986, and were licensed by the UK home office under Project License PPL60/3615 and approved by the local ethics committee at the University of Aberdeen Rowett Institute of Nutrition and Health (Approval numbers: SA07/14E and SA08/17E).

### Animals

Rats used in the study were male F344/N, purchased from Harlan Sprague Dawley Inc. USA at approx. 4 weeks old and were acclimatised for at least one week in a 12 h∶12 h light dark photoperiod. In all rat experiments, apart from room lighting changes, all other environmental conditions were the same; temperature was 21°C +/−2°C, food (CRM (P) Rat and Mouse Breeder and Grower, standard pelleted diet (Special Diet Services, Witham, Essex, UK)) and water were provided *ad libitum*. In all experiments where rats were moved into new photoschedules, they were transferred to rooms with the original lights-on time, thus for the SD or SD10∶14 rats, the photoperiods were shortened by advancing the lights off time.

### Rats for Microarray, gene expression and thyroid hormone assay

Rats were randomly divided into weight matched groups, housed singly and transferred from 12 h∶12 h light:dark into LD (16 h∶8 h, light:dark) or SD (8 h∶16 h, light:dark) photoperiod rooms. For time course analysis, rats were divided into six weight-matched groups (n = 8–10 for *in situ* hybridization, n = 6 for thyroid hormone assay and n = 4 for microarray) with 3 groups assigned to LD and 3 to SD. Food intakes and body weights were measured and recorded weekly and bi-weekly respectively where possible. After the appropriate number of days, one group of rats from each photoperiod was killed at ZT3 using isoflurane inhalation and decapitation. Trunk blood was collected into 15 ml tubes for serum preparation. Brains were immediately removed and frozen on dry-ice and then serum and brains were stored at −80°C.

### Photoperiod suppression by SD and daily melatonin injections

After acclimatization rats were housed initially in a LD photoperiod of 14 h∶10 h light:dark (LD14∶10) for 13d. Food intakes and body weights were recorded daily and 3x per week, respectively. Melatonin was administered according to Heideman *et al* [12]. For both the 3 and 14d duration experiments, rats were divided into 3 weight matched groups (n = 8). One LD14∶10 group was injected daily with melatonin (100 µg of melatonin dissolved in 0.1 ml of 10% ethanol and 90% physiological saline; delivered subcutaneously) whilst LD14∶10 and short day photoperiod (10 h∶14 h light:dark, SD10∶14) control groups were injected daily with vehicle (0.1 ml of 10% ethanolic saline). Injections were carried out 1 hour before lights off and continued for 3d or 14d at which time rats were anaesthetised using isoflurane inhalation and decapitated during the mid-light phase (8 hrs after lights on for LD and 4 hrs after lights on for SD). Brains were immediately removed and frozen on dry-ice and then stored at −80°C. Testes were dissected and weighed.

### Photoperiod induction experiment

Rats were initially exposed to SD10∶14 for 13d. Rats were divided into weight matched groups (n = 8). Two groups remained in SD10∶14, while 2 groups were transferred to LD14∶10. Food intakes and body weights were recorded daily and 3x per week, respectively. After 3d, one SD and one LD group was killed and the remaining rats were killed after 14d during the mid-light phase and tissues collected as described above.

### Microarray analysis

Hypothalamic blocks encompassing the ARC were dissected whilst frozen and total RNA was extracted for microarray analysis and gene cloning. For microarray, 4 hypothalamic blocks were used for each time point in both photoperiods. The total RNA extracts from individual blocks were applied to separate arrays, thus 24 arrays were used for comparisons. Microarray analysis was done on Affymetrix rat 230-2.0 Genechips by ServiceXS. Data from Service XS, in the CEL file format (containing the raw signal intensities) were transferred to the MadMax website (https://madmax.bioinformatics.nl) where data was analysed using the statistical programming language R [35] and R-libraries offered by the Bioconductor project [36]. The data were normalized using Bioconductor and a GCRMA method, a Robust Multiarray Analysis with correction for the G:C content of the oligos, and then statistically analysed using the Limma package, which allowed identification of the most differentially expressed genes between different conditions, i.e. photoperiods and time using a nominal *p*-value <0.05 to represent statistical significance. The MIAME compliant data for experimental conditions and data sets were submitted to the Gene Expression Omnibus and are accessible with the accession number GSE27926 (http://www.ncbi.nlm.nih.gov/geo/).

### In situ hybridization

The expression distribution and mRNA levels of a selection of thyroid-signalling related genes were analysed in forebrain coronal sections. These were cut and analysed by *in situ* hybridization techniques previously described in detail [11]. Riboprobe templates were prepared as described earlier for the genes *TRH* [37], *TSHβ* [38], *Dio2* and *Dio3* [2], *MCT-8* [22] and *GHRH* [11]. Riboprobe templates were prepared for *GALP, CGA* and *Oatp1c1* by amplifying cDNAs from F344/N rat hypothalamus and then cloning *Oatp1c1* and *GALP* into pSC-B-amp/kan (Agilent Technologies UK Ltd, Edinburgh, UK) and *CGA* into pCR-Blunt 4 TOPO (Invitrogen, Paisley, UK). A *TSH-receptor (TSH-R)* riboprobe was amplified from mouse cDNA and cloned into pGEM-T-easy (Promega, Southampton, UK). Oligonucleotide primers for *GALP* were based on rat *GALP* (GenBank accession no. NM_033237), amplifying a 331 bp DNA fragment between bases 99–429. For *GALP*, forward primer was, 5′-TGTATGCCGTGCTTTTCCAGTTC and reverse primer was, 5′-CTATGCGCAGATCTCCAGTCCTC. Oligonucleotide primers for *CGA* were based on rat *CGA* (GenBank accession no. NM_053918), amplifying a 455 bp DNA fragment between bases 1–455. For *CGA*, forward primer was 5′-CTGCCCACAACACATCCTTCC and reverse primer was 5′-CGCACGGGTCAGCAGTCG. Oligonucleotide primers for *Oatp1c1* were based on rat *Oatp1c1* (GenBank accession no. NM_053441), amplifying a 409 bp DNA fragment between bases 125–533. For *Oatp1c1*, forward primer was 5′-GAGGCCAACTGGAAAGGCGAAAGA and reverse primer was 5′-AAGACAGGGCACCCAAGAACAC. Oligonucleotide primers for *TSH-R* were based on mouse *TSH-R* (GenBank accession no. NM_011648), amplifying a 312 bp DNA fragment between bases 607–918. For *TSH-R*, forward primer was 5′- TCCAGGGCCTATGCAATGAAAC and reverse primer was 5′- CAGCCCGAGTGAGGTGGAGGAA. PCR amplifications of *GALP, Oatp1c1* and *CGA* were performed using KOD hotstart (Novagen, Merck Chemicals Ltd. Nottingham, U.K.) and for *TSH-R* using Go Taq Hotstart (Promega, Southampton, UK). PCR amplification conditions were denaturation for 1 cycle at 95°C for 2 minutes and then 35 cycles (*TSH-R*), 30 cycles (*GALP* and *Oatp1c1*) or 25 cycles (*CGA*) at 95°C for 20 sec (30 sec for *CGA* and *TSH-R*), annealing at 58°C for *GALP*, 55°C for *Oatp1c1* and *TSH-R*, and 56°C for *CGA* for 30 sec and extension at 70°C for 20 sec for *GALP* and *Oatp1c1* and 72°C for 30 sec for *CGA* and 45 sec for *TSH-R* with a 10 minutes extension at 72°C for *CGA* and *TSH-R*.

Sense riboprobes were synthesised from the complementary DNA template strands and generated no signals in the hypothalamus or *PT* (Figure S2). Autoradiographic images were quantified using Image-Pro Plus software version 7.0 (Media Cybernetics UK, Marlow, Buckinghamshire, UK), which computes the integrated optical density of the signal relative to a standard curve generated by ^14^C autoradiographic microscales (Amersham Pharmacia Biotech UK Ltd, Little Chalfont, Buckinghamshire, UK).

### T3/4 analysis

Intracellular hypothalamic T4 and T3 concentrations were measured by radio-immuno assay [39] following extraction of the tissues as previously described in detail [40]. In short, hypothalamic tissue blocks of an average of 49.1 mg each, pooled two by two, were homogenized in a methanol volume 3 times the tissue's weight. As individual internal recovery tracers, 1500–2000 cpm of outer ring labeled [^131^I]T3 and [^125^I]T4 were added. A volume of chloroform, twice the volume of methanol, was added. After centrifugation (15 min, 1900 g), the pellet was re-extracted in a mixture of chloroform and methanol (2∶1). Back-extraction into an aqueous phase (0.05% CaCl_2_) was followed by a re-extraction with a mixture of chloroform:methanol:0.05% CaCl_2_ (3∶49∶48) and this phase was further purified on Bio-Rad AG 1-X2 resin columns. The iodothyronines were eluted with 70% acetic acid, evaporated to dryness and resuspended in RIA buffer. Typical recoveries of extracted thyroid hormones ranged from 55 to 75% for T3 and from 40 to 60% for T4. The T3 RIA had a detection limit of 2 fmol and an intra-assay variability of 2.2%. The T4 RIA had a detection limit of 5 fmol and an intra-assay variability of 2.8%. For the T3 RIA cross-reactivity with T4 was 0.1–0.5%, whereas for the T4 RIA cross-reactivity with T3 was 3.5%. All samples were measured within a single assay.

### Statistics

Data were analysed by *t* tests and one or two-way ANOVA and repeated measures ANOVA, followed by Holm-Sidak method for pairwise comparisons as appropriate, using SigmaStat statistical software (Systat Software UK Ltd, Hounslow, London, UK). Results are presented as means ± SEM, and differences were considered significant at *p*<0.05.

## Supporting Information

Figure S1 **Paired testes weights of melatonin injected rats.** The paired testes weights did not show any significant difference with photoperiod or melatonin injections. Data are mean ± SE.
(TIF)

Figure S2 **Autoradiograph images using sense riboprobes.** Sense riboprobes showed no detectable hybridization signals in the rat hypothalamic and *PT* regions. Sense riboprobes used were; (A) *TRH*, (B) *TSHβ*, (C) *CGA*, (D) *Dio2*, (E) *Dio3*, (F) *MCT-8*, (G) *Oatp1c1*, (H) *NPY*, (I) *GALP*, and (J) *GHRH*. Scale bar  =  1.0mm for all images.
(TIF)

## References

[1] Hanon EA, Lincoln GA, Fustin JM, Dardente H, Masson-Pevet M (2008). Ancestral TSH mechanism signals summer in a photoperiodic mammal.. Curr Biol.

[2] Barrett P, Ebling FJ, Schuhler S, Wilson D, Ross AW (2007). Hypothalamic thyroid hormone catabolism acts as a gatekeeper for the seasonal control of body weight and reproduction.. Endocrinology.

[3] Yoshimura T, Yasuo S, Watanabe M, Iigo M, Yamamura T (2003). Light-induced hormone conversion of T4 to T3 regulates photoperiodic response of gonads in birds.. Nature.

[4] Dardente H, Wyse CA, Birnie MJ, Dupre SM, Loudon AS (2010). A molecular switch for photoperiod responsiveness in mammals.. Curr Biol.

[5] Nakao N, Ono H, Yamamura T, Anraku T, Takagi T (2008). Thyrotrophin in the pars tuberalis triggers photoperiodic response.. Nature.

[6] Yasuo S, Watanabe M, Nakao N, Takagi T, Follett BK (2005). The reciprocal switching of two thyroid hormone-activating and -inactivating enzyme genes is involved in the photoperiodic gonadal response of Japanese quail.. Endocrinology.

[7] Ono H, Hoshino Y, Yasuo S, Watanabe M, Nakane Y (2008). Involvement of thyrotropin in photoperiodic signal transduction in mice.. P Natl Acad Sci USA.

[8] Revel FG, Saboureau M, Pevet P, Mikkelsen JD, Simonneaux V (2006). Melatonin regulates type 2 deiodinase gene expression in the Syrian hamster.. Endocrinology.

[9] Yasuo S, Watanabe M, Iigo M, Nakamura TJ, Watanabe T (2007). Differential response of type 2 deiodinase gene expression to photoperiod between photoperiodic Fischer 344 and nonphotoperiodic Wistar rats.. Am J Physiol Regul Integr Comp Physiol.

[10] Heideman PD, Sylvester CJ (1997). Reproductive photoresponsiveness in unmanipulated male Fischer 344 laboratory rats.. Biol Reprod.

[11] Ross AW, Johnson CE, Bell LM, Reilly L, Duncan JS (2009). Divergent regulation of hypothalamic neuropeptide Y and agouti-related protein by photoperiod in F344 rats with differential food intake and growth.. J Neuroendocrinol.

[12] Heideman PD, Bierl CK, Sylvester CJ (2001). Photoresponsive Fischer 344 Rats are reproductively inhibited by melatonin and differ in 2-[125I] lodomelatonin binding from nonphotoresponsive Sprague-Dawley rats.. J Neuroendocrinol.

[13] Kuramochi M, Kohno D, Onaka T, Kato S, Yada T (2005). Galanin-like peptide and ghrelin increase cytosolic Ca2+ in neurons containing growth hormone-releasing hormone in the arcuate nucleus.. Regul Pept.

[14] Cunningham MJ, Krasnow SM, Gevers EF, Chen P, Thompson CK (2004). Regulation of galanin-like peptide gene expression by pituitary hormones and their downstream targets.. J Neuroendocrinol.

[15] Park S, Peng XD, Frohman LA, Kineman RD (2005). Expression analysis of hypothalamic and pituitary components of the growth hormone axis in fasted and streptozotocin-treated neuropeptide Y (NPY)-intact (NPY+/+) and NPY-knockout (NPY-/-) mice.. Neuroendocrinology.

[16] Shearer KD, Goodman TH, Ross AW, Reilly L, Morgan PJ (2010). Photoperiodic regulation of retinoic acid signaling in the hypothalamus.. J Neurochem.

[17] Matzuk MM, Kornmeier CM, Whitfield GK, Kourides IA, Boime I (1988). The glycoprotein alpha-subunit is critical for secretion and stability of the human thyrotropin beta-subunit.. Mol Endocrinol.

[18] Yasuo S, Nakao N, Ohkura S, Iigo M, Hagiwara S (2006). Long-day suppressed expression of type 2 deiodinase gene in the mediobasal hypothalamus of the Saanen goat, a short-day breeder: implication for seasonal window of thyroid hormone action on reproductive neuroendocrine axis.. Endocrinology.

[19] Yasuo S, Yoshimura T, Ebihara S, Korf HW (2007). Temporal dynamics of type 2 deiodinase expression after melatonin injections in Syrian hamsters.. Endocrinology.

[20] Yasuo S, Yoshimura T, Ebihara S, Korf HW (2010). Photoperiodic control of TSH-beta expression in the mammalian pars tuberalis has different impacts on the induction and suppression of the hypothalamo-hypopysial gonadal axis.. J Neuroendocrinol.

[21] Friesema EC, Jansen J, Jachtenberg JW, Visser WE, Kester MH (2008). Effective cellular uptake and efflux of thyroid hormone by human monocarboxylate transporter 10.. Mol Endocrinol.

[22] Herwig A, Wilson D, Logie TJ, Boelen A, Morgan PJ (2009). Photoperiod and acute energy deficits interact on components of the thyroid hormone system in hypothalamic tanycytes of the Siberian hamster.. Am J Physiol Regul Integr Comp Physiol.

[23] Sugiyama D, Kusuhara H, Taniguchi H, Ishikawa S, Nozaki Y (2003). Functional characterization of rat brain-specific organic anion transporter (Oatp14) at the blood-brain barrier: high affinity transporter for thyroxine.. J Biol Chem.

[24] van der Deure WM, Hansen PS, Peeters RP, Kyvik KO, Friesema EC (2008). Thyroid hormone transport and metabolism by organic anion transporter 1C1 and consequences of genetic variation.. Endocrinology.

[25] Nakao N, Takagi T, Iigo M, Tsukamoto T, Yasuo S (2006). Possible involvement of organic anion transporting polypeptide 1c1 in the photoperiodic response of gonads in birds.. Endocrinology.

[26] Segerson TP, Kauer J, Wolfe HC, Mobtaker H, Wu P (1987). Thyroid hormone regulates TRH biosynthesis in the paraventricular nucleus of the rat hypothalamus.. Science.

[27] Chiamolera MI, Wondisford FE (2009). Minireview: Thyrotropin-releasing hormone and the thyroid hormone feedback mechanism.. Endocrinology.

[28] Ohtaki T, Kumano S, Ishibashi Y, Ogi K, Matsui H (1999). Isolation and cDNA cloning of a novel galanin-like peptide (GALP) from porcine hypothalamus.. J Biol Chem.

[29] Takatsu Y, Matsumoto H, Ohtaki T, Kumano S, Kitada C (2001). Distribution of galanin-like peptide in the rat brain.. Endocrinology.

[30] Larm JA, Gundlach AL (2000). Galanin-like peptide (GALP) mRNA expression is restricted to arcuate nucleus of hypothalamus in adult male rat brain.. Neuroendocrinology.

[31] Lawrence C, Fraley GS (2011). Galanin-like peptide (GALP) is a hypothalamic regulator of energy homeostasis and reproduction.. Front Neuroendocrinol.

[32] Shahab M, Cunningham MJ, Steiner RA, Plant TM (2005). Galanin-Like peptide elicits a robust discharge of growth hormone in the rhesus monkey (Macaca mulatta).. Neuroendocrinology.

[33] Rich N, Reyes P, Reap L, Goswami R, Fraley GS (2007). Sex differences in the effect of prepubertal GALP infusion on growth, metabolism and LH secretion.. Physiol Behav.

[34] Gahete MD, Duran-Prado M, Luque RM, Martinez-Fuentes AJ, Quintero A (2009). Understanding the multifactorial control of growth hormone release by somatotropes: lessons from comparative endocrinology.. Ann N Y Acad Sci.

[35] Ihaka R, Gentleman R (1996). R: A language for data analysis and graphics.. J Comput Graph Stat.

[36] Gentleman RC, Carey VJ, Bates DM, Bolstad B, Dettling M (2004). Bioconductor: open software development for computational biology and bioinformatics.. Genome Biol.

[37] Ebling FJ, Wilson D, Wood J, Hughes D, Mercer JG (2008). The thyrotropin-releasing hormone secretory system in the hypothalamus of the Siberian hamster in long and short photoperiods.. J Neuroendocrinol.

[38] Sanchez E, Singru PS, Wittmann G, Nouriel SS, Barrett P (2010). Contribution of TNF-alpha and nuclear factor-kappaB signaling to type 2 iodothyronine deiodinase activation in the mediobasal hypothalamus after lipopolysaccharide administration.. Endocrinology.

[39] Darras VM, Huybrechts LM, Berghman L, Kuhn ER, Decuypere E (1990). Ontogeny of the effect of purified chicken growth hormone on the liver 5′ monodeiodination activity in the chicken: reversal of the activity after hatching.. Gen Comp Endocrinol.

[40] Reyns GE, Janssens KA, Buyse J, Kuhn ER, Darras VM (2002). Changes in thyroid hormone levels in chicken liver during fasting and refeeding.. Comp Biochem Physiol B Biochem Mol Biol.

