# Supplementary figures and images for: Thyroid Hormone Signalling Genes Are Regulated by Photoperiod in the Hypothalamus of F344 Rats
Source: PLoS One. 2011 Jun 22;6(6):e21351. doi: 10.1371/journal.pone.0021351 (PMC3120865; doi:10.1371/journal.pone.0021351)

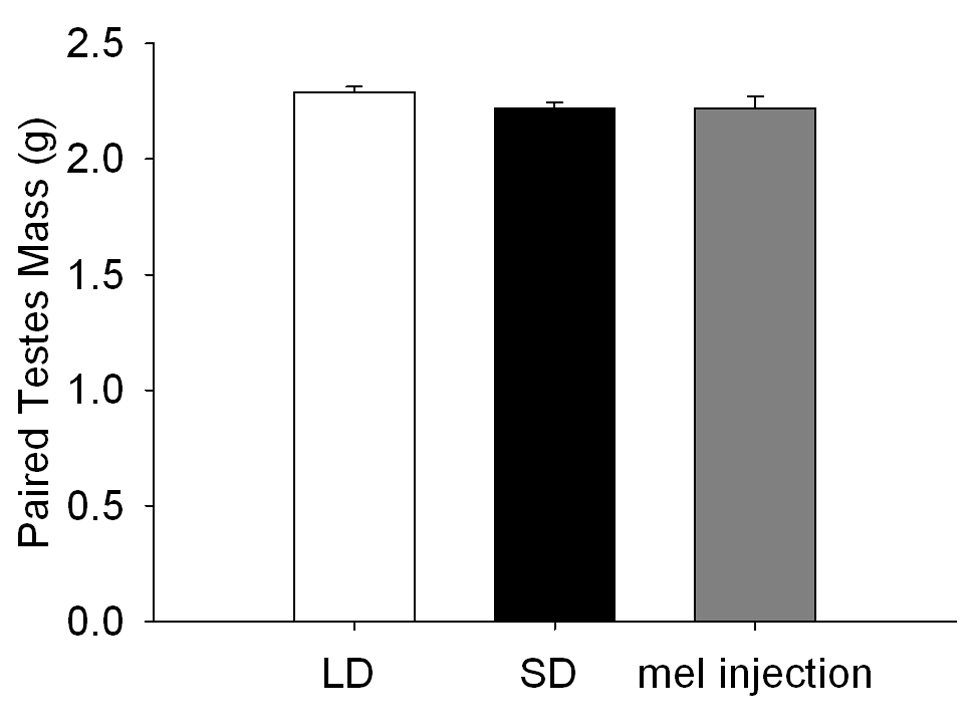

Supplement: Figure S1 — Paired testes weights of melatonin injected rats. The paired testes weights did not show any significant difference with photoperiod or melatonin injections. Data are mean ± SE. (TIF) [file pone.0021351.s001.tif]

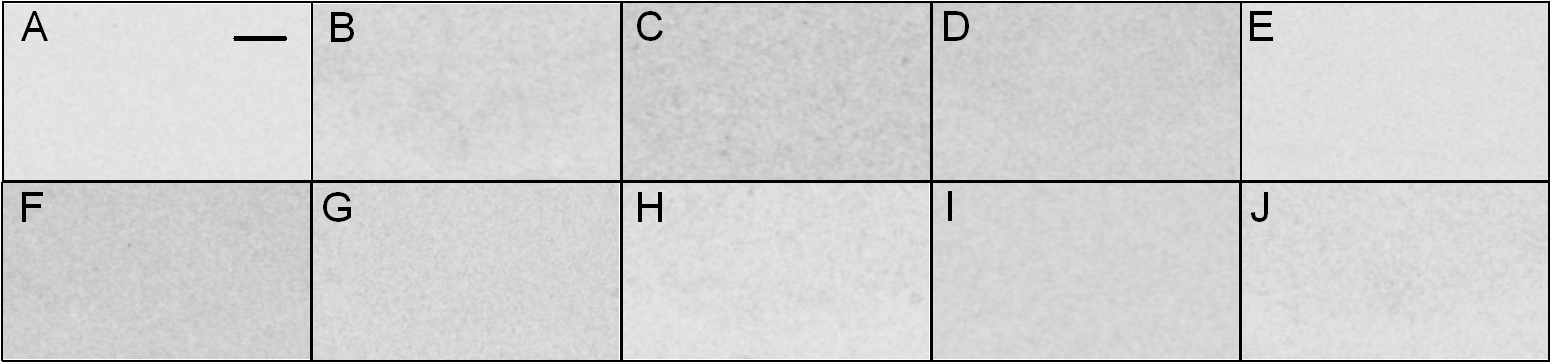

Supplement: Figure S2 — Autoradiograph images using sense riboprobes. Sense riboprobes showed no detectable hybridization signals in the rat hypothalamic and PT regions. Sense riboprobes used were; (A) TRH, (B) TSHβ, (C) CGA, (D) Dio2, (E) Dio3, (F) MCT-8, (G) Oatp1c1, (H) NPY, (I) GALP, and (J) GHRH. Scale bar = 1.0mm for all images. (TIF) [file pone.0021351.s002.tif]
